# Autoantibodies in Chronic Obstructive Pulmonary Disease

**DOI:** 10.3389/fimmu.2018.00066

**Published:** 2018-01-25

**Authors:** Lifang Wen, Susanne Krauss-Etschmann, Frank Petersen, Xinhua Yu

**Affiliations:** ^1^Xiamen-Borstel Joint Laboratory of Autoimmunity, Medical College of Xiamen University, Xiamen, China; ^2^Priority Area Asthma and Allergy, Research Center Borstel, Airway Research Center North (ARCN), German Center for Lung Research (DZL), Borstel, Germany; ^3^Institute of Experimental Medicine, Christian-Albrechts-University of Kiel, Kiel, Germany

**Keywords:** autoimmunity, autoantibodies, chronic obstructive pulmonary disease, biomarkers, emphysema, experimental models, pathogenesis

## Abstract

Chronic obstructive pulmonary disease (COPD), the fourth leading cause of death worldwide, is characterized by irreversible airflow limitation based on obstructive bronchiolitis, emphysema, and chronic pulmonary inflammation. Inhaled toxic gases and particles, e.g., cigarette smoke, are major etiologic factors for COPD, while the pathogenesis of the disease is only partially understood. Over the past decade, an increasing body of evidence has been accumulated for a link between COPD and autoimmunity. Studies with clinical samples have demonstrated that autoantibodies are present in sera of COPD patients and some of these antibodies correlate with specific disease phenotypes. Furthermore, evidence from animal models of COPD has shown that autoimmunity against pulmonary antigens occur during disease development and is capable of mediating COPD-like symptoms. The idea that autoimmunity could contribute to the development of COPD provides a new angle to understand the pathogenesis of the disease. In this review article, we provide an advanced overview in this field and critically discuss the role of autoantibodies in the pathogenesis of COPD.

## Introduction

Chronic obstructive pulmonary disease (COPD) is a major public health problem affecting more than 200 million people worldwide and leading to millions of death annually (1). COPD patients suffer from a progressive and not fully reversible airflow limitation. Pathologically, COPD is characterized by persistent pulmonary inflammation, obstruction of the small airways (obstructive bronchiolitis) and structural changes of the airways (emphysema) (2). It is widely accepted that COPD is triggered by inhaled toxic gases and particles. However, the pathogenesis of COPD remains largely unclear (2, 3).

Cigarette smoking (CS) is the major etiologic and risk factor for COPD (4, 5), and smoking cessation is beneficial for patients in terms of lung function (6). However, smoke cessation does not attenuate the pulmonary inflammation once COPD is established in patients (7, 8). The persistence of the pulmonary inflammation after smoking cessation and the presence of well-organized lymphoid follicles around small airways and lung parenchyma of COPD patients (9–11) implicate that there are memory adaptive immune responses to non-cigarette antigens, such as autoantigens, commensal microbiota, and infectious pathogens (8, 12, 13). Among these candidates, autoantigens are of specific interest because both clinical and experimental evidence suggest that CS is capable of triggering autoimmunity. Thus, the exploration of the contribution of autoimmune responses to the development of COPD could provide a new angle for understanding the pathogenesis of this disease.

In 2002, Cosio et al. proposed a novel concept that COPD could be considered as an autoimmune disease triggered by smoking (14). In 2007, Lee et al. reported that emphysema is characterized by the presence of humoral and cellular autoimmune responses against elastin, an extracellular protein important for lung integrity (12), for the first time showing evidence for a role of autoimmunity in COPD pathogenesis. Thereafter, many efforts have been made to determine the role of autoimmunity in the development of COPD by the use of clinical samples and animal models. In this review article, we aim to summarize recent advances in this field, discuss the contribution of autoimmunity to COPD, and outline prospects for future research. Since the role of self-reactive T cells in COPD has been reviewed elsewhere (14–16), we focus here on autoantibodies in COPD and its animal models.

## Autoantibodies in COPD Patients

### Presence of Autoantibodies in COPD

According to their targets, studies investigating the presence of autoantibodies in COPD can be roughly categorized into two groups, those addressing autoantibodies against undefined and those addressing defined antigens (Table 1).

**Table 1:** Summary of autoantibodies in COPD.

Autoantigen			Method	COPD vs control	Association with disease parameters	Reference
Tissues and cells	Tissue	Rodent tissue	IIF	Increased	ATS/ERS stage and DLCO	(19)
		Human lung tissue	DIH	Increased	–	(17)
		Human lung tissue	IIH	Increased	Emphysema	(18)
	Epithelial	HEp-2 cells	IIF	Increased	No	(17)
		primary PAEpC	IIF	Increased	–	(17)
		HBEC	IIF	Increased	GOLD stages	(22)
	Endothelial	primary PAEdC	IIF	Increased	–	(17)
		HUVEC	ELISA	Increased	No	(20)
		HUVEC	ELISA	Increased	–	(21)
Defined autoantigens	ECM proteins	Elastin	ELISA	Increased	–	(12)
			PA	Increased	–	(18)
			ELISA	No difference	–	(25)
			ELISA	No difference	–	(23)
			ELISA	No difference	–	(26)
			ELISA	No difference	–	(24)
			ELISA	Decreased	Emphysema and GOLD stage	(27)
			ELISA	Decreased	–	(28)
		Collagen I	PA	Increased	–	(18)
			ELISA	No difference	–	(23)
			ELISA	No difference	–	(12)
			ELISA	Decreased	–	(25)
		Collagen II	PA	Increased	Emphysema	(18)
			ELISA	No difference	–	(25)
		Collagen IV	PA	Increased	–	(18)
			ELISA	No difference	–	(25)
		Aggrecan	PA	Increased	Emphysema	(18)
	Alveolar cellular proteins	Cytokeratin 18	WB	Increased	FEV1(L) and FEV1 (% predicted)	(29)
			ELISA	Increased	GOLD stage	(30)
			ELISA	No difference	–	(25)
		Cytokeratin 19	ELISA	Increased	GOLD stage	(30)
	Immune molecules	Soluble CD80	ELISA, WB	Increased	GOLD stage	(38)
		αB-crystallin	ELISA	Increased	No association	(37)
		β2-Microglobulin	PA	Increased	–	(18)
	Neo-autoantigen	CCP	ELISA	Increased	No association	(35)
			ELISA	No difference	–	(33)
			ELISA	No difference	–	(34)
			ELISA	No difference	–	(28)
		CFFCP	ELISA	Increased	No association	(34)
		MCV	ELISA	No difference	No association	(28)
		CMP	ELISA	Increased	FEV1 (% predicted) and GOLD stages	(21)
	Autoantigens in common autoimmune disease	Nuclear	IIF	Increased	No association	(19)
			IIF	Increased	No association	(39)
			IIF	No difference	–	(33)
			IIF	No difference	–	(26)
		Cardiolipin, CENP-B, cytochrome *C*, DGPs, H1, H2A, H2B, histone, JO-1, La/SS-B, LC1, PL-12, PL-7, Ro52 (SSA), thyroglobulin, topoisomerase, snRNP-68, U1-snRNP-BB, and U1-snRNP-A	PA	Increase	Emphysema	(18)

*HEp-2 cells, hepatoma cell; primary PAEpC, primary pulmonary airway epithelial cells; HBEC, human bronchial epithelial cell; primary PAEdC, primary pulmonary artery endothelial cells; HUVEC, human umbilical vein endothelial cell; IIF, indirect immunofluorescence, DIH, direct immunohistochemistry; IIH, indirect immunohistochemistry; ELISA, enzyme-linked immunosorbent assay; WB, western blot; PA, protein array; ECM, extracellular matrix proteins; CCP, citrullinated peptides; CFFCP, chimeric citrullinated peptides of human fibrin and filaggrin; MCV, mutated citrullinated vimentin; DGPs, deamidated gliadin peptides; CMP, carbonyl-modified proteins; COPD, chronic obstructive pulmonary disease; ATS/ERS, American Thoracic Society/European Respiratory Society*.

The first group consists of studies in which autoantibodies against undefined autoantigens, such as tissues or cells were investigated. Given that the majority of autoantigens in COPD is probably unknown, determination of autoantibodies against lung tissue or its major cell types, e.g., epithelial or endothelial cells provides a proof-of-principle evidence for the presence of autoantibodies in this disease. In 2008, Feghali-Bostwick and colleagues detected IgG deposition within alveolar septa and small airway walls by immunohistochemical staining in six out of six of patients with severe COPD, but in none of six controls (17) indicating for the first time that anti-tissue antibodies are present in COPD patients. Such anti-tissue antibodies in COPD were also described in another study using indirect immunohistochemical staining in which Packard et al. showed that IgG from COPD patients have a higher binding capability to non-smoker lung tissue section than IgG from healthy smokers (18). In addition, anti-tissue antibodies in COPD could also be detected using rodent tissues as antigen (19) confirming the presence of anti-tissue antibodies.

Aside from studying anti-tissue autoantibodies, Feghali-Bostwick et al. went on to investigate autoantibodies more specifically directed against epithelial and endothelial cells (17). Their results showed that the prevalence of autoantibodies against epithelial cells, including both hepatoma (HEp-2) cell and primary pulmonary artery epithelial cells, is significantly higher in COPD patients as compared with smoker or non-smoker (17). In addition, 50% of the COPD patients are also characterized by autoantibodies against primary pulmonary artery endothelial cells (17). This study shows for the first time the presence of anti-epithelial and anti-endothelial autoantibodies in COPD patients. By using a cell-based enzyme-linked immunosorbent assay with human umbilical vein endothelial cells (HUVECs) as coated antigen, two independent research groups confirmed the presence of anti-endothelial IgG in COPD patients (20, 21). Recently, the presence of anti-epithelial IgG has also been shown in a study using human bronchial epithelial cells as target (22). Taken together, these studies demonstrate convincingly that autoantibodies against lung tissue as well as pulmonary epithelial and endothelial cells are detectable in patients with COPD.

In the second group of studies, autoantibodies against defined antigens suspected to be present in or functionally related to the disease were investigated. This group includes extracellular matrix (ECM) proteins, cellular proteins from pulmonary cells, neo-autoantigens, immune molecules, and autoantigens in common autoimmune diseases.

The main function of cellular matrix proteins is to maintain the structure and integrity of the lung. Since degradation of matrix proteins is a hallmark of emphysema, these proteins are promising candidates for autoantigens in COPD. As mentioned earlier, autoantibodies against elastin have been detected in emphysema patients, showing for the first time the presence of autoantibodies in COPD (12). However, beside one exception (18), all following studies failed to confirm levels of anti-elastin antibodies in COPD patients surmounting those of healthy controls (23–26). Moreover, results from two independent groups showed that levels of anti-elastin antibodies are even lower in COPD patients than in healthy controls (27, 28). Beside elastin, collagens have been also extensively investigated as candidate autoantigens for COPD. Using an autoantigen array with 70 proteins, Packard and colleagues showed that levels of autoantibodies reactive to a broad spectrum of self-antigens are significantly higher in COPD patients involving emphysema than in healthy controls, including autoantibodies against collagen I, collagen II, and collagen IV (18). However, similar to the findings for anti-elastase antibodies, these results were not confirmed by other groups (12, 23, 25), keeping this issue under debate. Moreover, autoantibodies against aggrecan, another ECM protein, have been detected in patients with COPD (18).

In addition to ECM proteins, cellular proteins from pulmonary cells have also been regarded as potential autoantigens in COPD. Using immunoblotting assays with lysates prepared from alveolar cells as targets, Kuo et al. showed that autoantibodies against multiple cellular antigens are more frequently present in sera of COPD patients than in controls (29). Among those antigens, a 45-kDa cellular protein was identified as cytokeratin 18 (CK-18), an intermediate filament protein located in the intracytoplasmic cytoskeleton of epithelial tissue. Although this interesting finding has not been observed in a report based on a small number of samples (25), it has been confirmed in a recent study where 228 COPD patients and 136 controls were included (30). Here, Xiong et al. could show that the levels of circulating IgG, IgA, and IgM autoantibodies against CK-18 are elevated in COPD patients as compared with healthy controls (30). Furthermore, they also demonstrated that COPD patient express autoantibodies against another intermediate filament protein, CK-19, suggesting another cellular autoantigen for the diseases (30).

Neo-autoantigens are antigens expressed under specific pathophysiological conditions and are not ubiquitously in our body (31). Well-known neo-autoantigens in autoimmune diseases such as rheumatoid arthritis (RA) are citrullinated peptides or proteins which are generated by a posttranslational modification process occurring under certain inflammatory conditions (32). Results from several studies using different citrullinated peptides or proteins as antigens have shown that the frequencies of sera with anti-citrullinated protein antibody (ACPA) are very low in both COPD patients and healthy controls and without significant difference between these two groups (33–35). When the concentrations of ACPA are used for comparison, levels of anti-cyclic citrullinated peptide antibodies (CCP2) have been shown to be higher in sera of COPD patients than controls in one study (35), which was not confirmed by three other groups (28, 33, 34). Carbonyl-modified proteins (CMP) represent another type of neo-autoantigen, which are investigated in COPD. Oxidants, a major constituent of cigarette smoke, can cause the formation of carbonyl adducts on proteins *in vivo* (36), making it conceivable that autoantibodies against CMP are generated in COPD patients. To verify this hypothesis, Kirkham et al. determined levels of autoantibodies against CMP in the sera of COPD patients and controls. They found that antibody titers against carbonyl-modified self-protein were significantly increased in patients with COPD as compared with controls (21) showing the presence of such autoantibodies.

Apart from molecules from cells residing in the lung, some molecules from immune cells have also been shown to act as autoantigens in COPD. In 2012, Cherneva and colleagues reported that concentrations of autoantibodies against αB-crystallin (HspB5), a marker of innate immune activation, were increased in patients with COPD (37). Notably, according to this study, those autoantibodies are also present in inflammatory lung diseases suggesting that they are not COPD specific. Very recently, Luo et al. investigated autoantibodies against a soluble form of CD80 (sCD80), a co-stimulatory molecule for T cell activation, in sera of patients with COPD (38). They found that serum levels of anti-sCD80 were higher in patients with COPD than in controls and were positively correlated to inflammatory cytokines, e.g., IL-6 and IL-8 (38). Another immune molecule, β2-microglobulin that is a component of MHC class I molecules, has also been identified as an autoantigen in COPD (18).

Finally, autoantigens that have already been described in some common autoimmune diseases have also been investigated in COPD. For example, using indirect immunofluorescence staining, two independent groups have demonstrated that antinuclear antibodies are more prevalent in patients with COPD than healthy controls (19, 39). Although this difference has not been found in two other studies using the same detection method (26, 33), a study using protein arrays carrying 70 different antigens has confirmed that sera of patients with emphysema have autoantibodies reactive to many common nuclear antigens (18). In this study, the reactivity of sera derived from patients suffering from systemic lupus erythematosus (SLE) and RA was analyzed in comparison. Interestingly, emphysema-associated COPD was characterized by a lower autoantibody reactivity than SLE, but a higher than RA (18), suggesting COPD is indeed associated with a substantial level of autoimmunity.

Taken together, a number of previous studies have demonstrated that autoantibodies are present in patients with COPD. However, their determination and visualization appear to be autoantigen and method depending.

### Does the Presence of Autoantibodies Correlate with Clinical Parameters in COPD?

Aside from demonstrating the mere presence of autoantibodies, their potential correlation with disease parameters is important for their clinical relevance as biomarkers and could provide further evidence for a role of autoantibodies in COPD. Therefore, some studies with well characterized patients investigated the correlation between autoantibodies and clinical parameters of COPD.

By comparing subgroups of COPD patients categorized by clinical parameters, Nunez and colleagues demonstrated that the prevalence of anti-tissue antibodies are significantly different among patients groups with various disease severity as indicated by American Thoracic Society/European Respiratory Society (ATS/ERS) stage or diffusing capacity of carbon monoxide (DLCO). Patients with more severe disease showed higher prevalence of anti-tissue antibodies (19), suggesting an association between the presence of anti-tissue antibodies and an increased disease severity. In another study, Packard and colleagues described that sera from COPD patients with emphysema showed a higher anti-tissue antibody reactivity than sera from COPD patients without emphysema, suggesting an association of this autoantibody with emphysematous disease.

Recently, an association between severity of COPD and anti-epithelial antibodies has been demonstrated by Cheng et al. (22). They found that the prevalence of both anti-epithelial IgG and IgA are elevated in patients with severe disease (GOLD stage III or IV) as compared with patients with milder symptoms (GOLD stage I or II) (22). However, the association of anti-epithelial antibodies with disease severity has not been observed in a study with a rather small number of patients (17). Based on these inconsistent results from current studies, it remains unclear whether anti-epithelial antibodies are indeed associated with severity of COPD.

The correlation with clinical parameters of COPD has also been shown for autoantibodies directed against some defined antigens. For example, in Packard’s study, they found that serum levels of autoantibodies against many antigens are significantly higher than in COPD patients without emphysema (18). Disease severity is also reported to be associated with autoantibodies against several other antigens. In 2010, Kuo et al. reported that levels of autoantibodies against CK-18 were inversely correlated with lung function parameters (29). This correlation is confirmed by investigations of Xiong et al. who showed that circulating levels of both anti-CK-18 and anti-CK-19 autoantibodies correlate significantly with the severity of the disease (30). Besides anti-CK-18 and anti-CK-19, two other autoantibodies have been reported to correlate with disease severity in COPD. While the presence of anti-sCD80 antibodies was shown to be associated with a high GOLD stage (38), autoantibodies against CMP correlated with a high GOLD stage and were inversely correlated with lung function parameters (21).

Notably, anti-elastin antibodies are also associated with disease parameters, but in contrast to all autoantibodies mentioned earlier, in the opposite direction where a lower antibody titer was associated with more severe disease and emphysema (27).

## Autoantibodies in Animal Models of COPD

Animal models are powerful research tools for investigating the pathogenesis of human diseases. For COPD, such models have been established in many species, including mice, rat, dog, monkey, and guinea pig (40). In consistence with findings in COPD patients, evidence from animal models also supports a role of autoantibodies in the pathogenesis of COPD, especially in those with emphysema (Table 2).

**Table 2:** Autoantibodies in animal models of COPD.

Animal models	Disease symptoms	Autoantibodies	Reference
CS-exposed mice	Pulmonary inflammation with macrophages and B cells	Anti-ECM	(41)
ECM-immunized mice	Pulmonary inflammation with macrophages	Anti-ECM	(41)
CS-exposed rat	emphysema	Anti- β2-AR	(42)
β2-AR immunized rat	Emphysema	Anti- β2-AR	(42)
HUVEC-immunized rat	Emphysema	Anti-HUVEC	(44)
IgG transfer-induced rat	Emphysema	Anti-HUVEC	(44)

*CS, cigarette smoking; ECM, extracellular matrix, β2-AR, β2-adrenergic receptor; HUVEC, human umbilical vein endothelial cell; COPD, chronic obstructive pulmonary disease*.

In 2010, Brandsma and colleagues reported that chronic CS-exposure leads to the pulmonary inflammation and production of autoantibodies against multiple ECM components in mice (41). To further explore the role of autoantibodies against ECM in this mouse model, the authors immunized mice with a mixture of lung ECM. As expected, mice immunized with ECM produced high levels of autoantibodies against different ECM components. In addition, the immunization alone increased the number of macrophage in the lung tissue suggesting a proinflammatory role of autoimmunity against ECM (41). Unfortunately, emphysema, which is associated with autoantibodies in human COPD, was not observed in their model disabling the evaluation of a potential association between autoimmunity against ECM and emphysema development (41). Very recently, a study investigating CS-induced rat model of COPD shed some new light on this field (42). In this study, Hu et al. reported that CS-exposed rats produce higher levels of autoantibodies against β2-adrenergic receptors (β2-ARs) than control rats (42). Moreover, autoantibodies against β2-ARs are associated with severity of CS-induced emphysema suggesting that the autoimmunity might be involved in the formation of emphysema. To confirm this notion, the authors immunized rats with the peptide of the second extracellular loop of β2-ARs which contains an epitope recognized by agnostic autoantibodies against β2-ARs (42, 43). Rat immunized with the β2-ARs peptide produces autoantibodies against β2-ARs and development of emphysema, confirming the role of autoimmunity against β2-ARs in pathogenesis of COPD-like disease (42). Therefore, evidence from CS-induced animal models demonstrates that CS-exposure triggers autoimmunity against multiple autoantigens in the lung and such autoimmunity contribute to COPD-like symptoms.

Beside CS-expose induced approaches, models induced by active immunization also provide evidence for a role of autoimmunity in the pathogenesis of COPD. In 2005, Taraseviciene-Stewart and colleagues reported a new animal model for COPD in rats (44). Rats immunized with HUVECs raised antibodies against HUVECs and developed emphysema. Interestingly, adoptive transfer of either serum or CD4+ T cells from the HUVECs-immunized rat into naive immunocompetent rats induced subsequent emphysema (44). These results suggest an autoimmune-driven mechanism beneath the disease manifestation in experimental COPD which involves both autoreactive T cells and autoantibodies.

## Conclusion and Perspectives

In summary, the abovementioned clinical and experimental studies provide some evidence for the role of autoantibodies in COPD. First, autoantibodies are present in both COPD patients and in corresponding animal models, particularly those involving emphysema. This notion is supported by the finding that severe COPD with emphysema is associated with HLA II alleles which is associated with most autoimmune diseases (45). Second, correlation between autoantibodies and disease severity has been described but appears not to be consistent among different studies. Third, autoantibodies in animal models of COPD are capable of inducing a COPD-like disease phenotype. Those evidence, alone with the fact that COPD patients are characterized by increased numbers of B cells, plasma cells, and B cell-rich lymphoid follicles that correlate directly with disease severity (11, 46), support a role of autoantibodies in the development of COPD. Besides humoral autoimmunity, it is important to mention that cellular autoimmunity might also contribute to COPD pathogenesis. As aforementioned, human autoreactive CD8+ and CD4+ T cells have been suggested to contribute to COPD (14–16). In addition, transfer of CD4+ T cells from the HUVECs-immunized rat is able to induce emphysema, supporting a role of autoreactive CD4+ T cells in experimental COPD (44).

With immunodeficient mice which lack both T- and B-cells, the role of adaptive immunity in experimental COPD can be explored. In 2010, Motz et al. reported that transfer of T cells from CS-exposed mice, but not T cells from fresh air-exposed mice, into Rag2−/− mice led to the development of pulmonary inflammation and emphysema in the recipient mice (47), suggesting that adaptive immune responses are capable to mediate COPD-like symptoms. However, by directly exposing immunodeficient mice to CS, two research groups have demonstrated that immunodeficient mice develop comparable COPD-like disease as wild-type controls mice (48, 49), indicating that adaptive immune responses are not required for CS-induced COPD in mice. Since autoimmunity is a part of adaptive immune responses, the abovementioned experimental evidence suggests that autoimmunity is potentially pathogenic but dispensable for CS-induced COPD in animals. However, since human differ from animals in many aspects, findings from animal models might not sufficiently reflect human diseases. Therefore, whether autoimmunity is a pathogenic or an indispensable event in the development of COPD remains elucidative.

In conclusion, previous studies have suggested a potential role of autoimmunity in COPD and its animal models, opening a new field for exploring the pathogenesis of the diseases. Further investigations in this field will not only help for understanding the pathogenesis of COPD but also help for both diagnostic and treatment of the disease.

## Author Contributions

LW was involved in search and identification of relevant literatures. XY and LW were involved in drafting the manuscript. SK-E and FP critically read the manuscript and contributed with significant revision.

## Conflict of Interest Statement

The authors declare that the research was conducted in the absence of any commercial or financial relationships that could be construed as a potential conflict of interest.
